# Biodegradation of Conventional and Emerging Pollutants

**DOI:** 10.3390/molecules25051186

**Published:** 2020-03-06

**Authors:** Łukasz Chrzanowski, Łukasz Ławniczak

**Affiliations:** Institute of Chemical Technology and Engineering, Poznan University of Technology, Berdychowo 4, 60-965 Poznan, Poland

The concerns associated with the contamination of the environment remain a topic of great importance and growing interest. This is clearly evidenced by the shift of global trends towards sustainable development, the implementation of technical solutions which uphold the rules of Green Chemistry, high market demand for natural and bio-based products and legal changes in the regulation of hazardous waste. The notable increase of environmental awareness in social, academic and industrial sectors has contributed to an adamant will to instil appropriate changes. Nevertheless, there is a need for corresponding guidelines which would set a path for future considerations and indicate the first crucial steps which need to be taken.

This special issue entitled “Biodegradation of Conventional and Emerging Pollutants” has been prepared with the intent to elucidate the current state of the art regarding both well-known pollutants as well as compounds which may potentially become a hazard in the future. The issue includes seven original research articles and five reviews which together form an interesting combination of variable topics. Covering a wide spectrum of subjects—from heavy metals and hydrocarbons, through additives and heavy-duty protective agents to pharmaceuticals and pesticides—the Special Issue includes in-depth analyses regarding the environmental fate and impact of different pollutants, provides novel approaches to monitoring of their content, outlines the limitations of our current knowledge and indicates areas which require further improvement.

Dzionek et al. [1] reported the biodegradation of naproxen, a common nonsteroidal anti-inflammatory drug, using a system which consisted of trickling filters filled with microflora from a functioning wastewater treatment plant and supplemented with *Bacillus thuringiensis* cells immobilized on loofah sponge in. The studies indicated that naproxen caused a decrease of biodiversity in the trickling filter population, even after short-term exposure. Introduction of the immobilized *B*. *thuringiensis* cells allowed to achieve an efficient biodegradation rate of naproxen (up to 90% removal after 15 days with an initial concentration of 1 mg/L); however, it also affected the structure of the microbial community. Overall, the presented approach is a promising solution which may be applied in wastewater treatment plants to improve the treatment of naproxen and potentially, the related members of nonsteroidal anti-inflammatory drugs.

Immobilization is also the topic of the study by Tavares et al. [2]. In the framework of this report, a chemometric approach to the optimization of the immobilization of horseradish peroxidase using Δ-FeOOH as a carrier was described. Various factors, such as enzyme/iron oxide hydroxide nanoparticles ratio, pH, temperature and time, were analysed using a factorial design. The data were used to produce the immobilized enzymes, which were then subjected to a detailed characterization including X-ray powder diffraction, Fourier transform infrared spectroscopy and scanning electron microscopy in order to evaluate the changes in the morphology of the material. The study also includes a practical assessment of the obtained biocatalyst during the removal of ferulic acid as a model contaminant. The findings reveal that the biocatalyst obtained using optimal process parameters exhibited high removal efficiency of ferulic acid, which indicates that this approach is justified and may potentially be promising for the design of other immobilized enzymes.

Steliga et al. [3] presented a practical approach to bioremediation of soils contaminated with petroleum hydrocarbons and polychlorinated biphenyls (PCBs). The study presents a comparison of primary biodegradation and mineralization rates in case of treatment with bacterial monocultures (*Mycolicibacterium frederiksbergense* IN53, *Rhodococcus erythropolis* IN129 and *Rhodococcus* sp. IN306) as well as a mixed culture which included all three strains. The conducted analysis indicated various biodegradation capabilities of the studied bacterial strains. *Rhodococcus* sp. IN306 was the most efficient PCBs degrader (removal at 54%) whereas *Mycolicibacterium frederiksbergense* IN53 exhibited the highest biodegradation efficiency in case of petroleum hydrocarbons (37%). In case of a model system which consisted of sterile soil contaminated with hydrocarbons and PCBs, the mixed culture exhibited the best treatment efficiency. This was further confirmed during ex situ treatment of soil contaminated with aged petroleum compounds and spent transformer oil. Inoculation of the prism with the mixed culture allowed to decrease the hydrocarbon and PCBs content after 6 months by 71% and 85%, respectively, which allowed to notably decrease the phytoxicity of the treated soil. These findings indicate the feasibility of using multiple degraders to enhance the removal of recalcitrant compounds.

The topic of hydrocarbon removal is also elucidated in the study of Brzeszcz et al. [4]. The research was focused on the comparison of efficiency of biostimulation and bioaugmentation approaches in the removal of aliphatic and aromatic hydrocarbons from historically polluted soil. The bioaugmentation strategy involved two variants, an unidentified C1 community with randomly selected degraders and a C2 mixed culture with defined members. Interestingly, the results of the final biodegradation efficiency after 60 days indicated that the biostimulation approach was inferior (removal at 35%) compared to bioaugmentation. In the latter case, the use of the defined C2 mixed culture allowed to achieve improved removal of both aliphatic (87%) and aromatic (85%) hydrocarbons compared to the random C1 community (removal at 70% and 65%, respectively). The quality of the treated soil was notably improved as confirmed by toxicity assays using various indicator organisms. In-depth metagenomic analyses using Illumina high-throughput sequencing indicated that although both bioaugmentation approaches did not allow to ensure long-term stability of the introduced degraders, the C2 mixed culture was slightly more resistant to competition from autochthonous microbiota compared to the C1 community, which may explain its superior performance.

The study of Staninska-Pięta et al. [5] underlines the synergistic effects of mixed hydrocarbon and heavy metal contaminations, with special regard to the microbial secretion of exopolysaccharides (EPS) as a response to such stress factors. The presented findings confirm the deleterious effect of heavy metals on the structure of a microbial community, as indicated by the decrease of its biodiversity and metabolic activity. Interestingly, despite the negative impact of heavy metals, an increase of the polycyclic aromatic hydrocarbon degradation rate (by approx. 17%) was observed. Based on the assessment of the estimated number of genes, it was also established that the biodegradation pathway of polycyclic aromatic hydrocarbons is correlated with the synthesis of exopolysaccharides. Furthermore, the studies indicated that the analysed were predominant in microorganisms belonging to the *Burkholderiales* order, which suggests that they may be applied in bioremediation of areas co-contaminated by hydrocarbons and heavy metals.

Linhartová et al. [6] elucidated the biodegradation of two antimicrobial agents used in dental care products: chlorhexidine and octenidine. The experiments were carried out using *Irpex lacteus* and *Pleurotus ostreatus* white-rot fungi species. The obtained results indicated that among the two studied compounds, chlorhexidine was more susceptible to biodegradation with a removal rate of 70% after 21 days. In contrast, octenidine was more resistant, with an overall biodegradation efficiency equal to approx. 48%. The authors highlighted a very important issue associated with strong sorption of the analysed compounds on fungal biomass, which rendered their analysis challenging. It should also be noted that while primary biodegradation of both studied compounds was confirmed by the presence of their respective metabolites, the complete decomposition of both compounds could not be achieved, which highlights their recalcitrance and indicates that they may accumulate in the environment.

The investigation carried out by Tsai et al. [7] highlights the issue of contamination of meat products with phthalates and presents the results of analyses of 30 pork and 30 chicken meat samples carried out using liquid chromatography–tandem mass spectrometry. The samples were investigated with regard to the presence of the following compounds: diisononyl phthalate, diisodecyl phthalate, benzyl butyl phthalate, di-2-ethylhexyl phthalate (DEHP), and di-n-butyl phthalate. The results suggested that DEHP was the major contaminant, which was present in 8% of the studied meat samples and ranged from 0.62 to 0.80 mg/kg in pork and from 0.42–0.45 mg/kg in chicken meat. While the estimated risks associated with pollution of the tested samples was low, the study suggests the necessity to include efficient phthalate monitoring programs in order to avoid harmful effects and control the chronic exposure levels.

Lin et al. [8] prepared a comprehensive review regarding the degradation of methomyl in aqueous and terrestrial environments. As a commonly used pesticide which belongs to the carbamate group of compounds, methomyl is a xenobiotic that may be directly introduced to the environment. The review summarizes its fate in water and soil ecosystems along with its toxic effects towards various organisms. A comparison of possible removal methods with a strong emphasis on biodegradation approaches has been presented. Moreover, the most common biodegradation pathways of methomyl were presented and the most frequently identified metabolites were discussed. A summary of the most efficient methomyl degraders has also been included, with a detailed description of associated genes as well as enzymes responsible for its breakdown. The authors indicate the need for further research in this area, especially in terms of metagenomics analyses which may help understand the development of novel catabolic pathways.

The review by Luchnikova et al. [9] is focused on the possible use of microbial conversion processes to utilize resin acids, which are a common contaminant originating from the pulp and paper industry. The authors highlighted the pathways which result in the introduction of resin acids into the environment and discuss their toxicity as well as other negative effects associated with their presence. The main topic is associated with the biodegradation of resin acids, with a detailed presentation of pathways and possible products. In the following chapter, the possible application of bioconversion processes is discussed and the possible added-value bioactive products are presented. A list of strains which are capable of efficient conversion of resin acids (using dehydroabietic acid as an example) is of particular interest. The review presents promising opportunities which may potentially allow to transform harmful waste into valuable products which may be used in the industrial biotechnology sector.

Cao et al. [10] indicated the benefits of using digital PCR as an efficient tool for monitoring of biodegradation processes. The study provided a comprehensive overview regarding the application of two techniques, endpoint PCR and real-time quantitative PCR, for evaluating the progress of biodegradation. Then, the advantages of the novel solution, digital PCR, were discussed in relation to the previously described PCR protocols. The review expands to a valuable summary of applications of digital PCR in studies focused on evaluating the abundance of specific microbial taxa in a given populations, quantification of specific functional genes in a studied pool and assessment of gene expression, specifically in case of biodegradation assays. The review concludes with a list of potential limitations and highlights the chemical inhibition associated with the complexity of the studied microbial populations, the accuracy of the method, abundance of genes used for quantification as well as the analysis costs as the major factors which will determine the practical applicability of this protocol.

The fate of hydrocarbons in the environment is summarized in the review prepared by Truskewycz et al. [11]. The authors provide a detailed discussion regarding the possible interactions of petroleum-derived compounds with both abiotic and biotic elements. The changes of concentration of hydrocarbons during their introduction into the environment as a result of volatilization, dissolution, sorption and desorption processes were presented. The potential negative effects associated with the presence of hydrocarbons towards microbial populations were also elucidated. Afterwards, the process of natural attenuation was described, with particular emphasis on the factors which affect its final efficiency, namely the content of nutrients, salinity and the moisture level. One of the finest aspects of the review is the emphasis of the importance of mutual synergistic interactions among the species of degraders as a crucial trait for efficient biodegradation of hydrocarbon pollutants. This combined with a profound summary of hydrocarbon decomposition processes make for a fine lecture.

In contrast, the review by Ławniczak et al. [12] provided fundamental knowledge regarding the interactions between microorganisms and petroleum hydrocarbons, starting with the origin of crude oil and moving to practical aspects of bioremediation protocols. A brief description of the mutual relationship between petroleum and microbiota, which includes both dissipation of hydrocarbons as a source of carbon and energy as well as their production by various organisms, was presented initially. The description of a simple experiment with bacterial biofilms, which perfectly reflects the most relevant limitations of hydrocarbon biodegradation processes, is perhaps the most distinctive part of the review. Afterwards, a summary of strategies focused on enhancement of biodegradation efficiency was elucidated based on recent literature reports. The overview mainly included biostimulation, bioaugmentation and surfactant/biosurfactant-assisted biodegradation approaches and emphasized the limitations as well as crucial findings which may allow to improve the currently employed environmental clean-up practices. The final part of the review is a feasibility-based benchmark of the previously described strategies and includes basic guidelines which may increase the odds of successful bioremediation.

The articles form a comprehensive and concise summary of both advantages and shortcomings associated with the treatment of corresponding pollutants. Furthermore, they provide a solid base for future discussions regarding the improvement of currently employed environmental protocols. We wish to express our gratitude to all the Authors for their impactful contribution as well as all the Reviewers, who immensely helped in ensuring high scientific quality of the content. Finally, we hope that this Special Issue will be an interesting and insightful and inspiring lecture for all the Readers!
